# Alterations in lower-extremity sagittal plane joint moments due to experimental knee pain and effusion during walking

**DOI:** 10.1186/1757-1146-7-S1-A63

**Published:** 2014-04-08

**Authors:** Jihong Park, Devin C Francom, Matthew K Seeley, J Ty Hopkins

**Affiliations:** 1 Department of Sports Medicine, Kyung Hee University, Yongin, Korea; 2 Department of Applied Mathematics and Statistics, University of Santa Cruz, Santa Cruz, USA; 3 Department of Exercise Sciences, Brigham Young University, Provo, UT, USA

## Purpose

To examine acute alterations in lower-extremity sagittal plane joint moments due to isolated and/or combined experimental knee joint pain and effusion during walking.

## Methods

Nineteen able-bodied subjects walked four different conditions (control, effusion, pain, and pain+effusion), with a week between each condition. We used previously-used injury models of pain [1] and joint effusion [2] to the right side of the knee. The control condition consisted of no injection. For each condition, subjects completed three walking trials at three times: precondition (prior to injection(s)), condition (3 minutes post injection(s)), and postcondition (30 minutes post injections). We used a standard inverse dynamics approach (combining high speed video, ground reaction force, and anthropometric data) to estimate sagittal-plane, net, internal, joint moment for the hip, knee, and ankle during walking. A functional analysis of variance (FANOVA) approach was used to compare the aforementioned joint moment between conditions. This statistical approach allowed us to evaluate when differences exist, across the entire stance phase of gait, as well as the magnitude of the detected differences.

## Results

**Figure 1:**
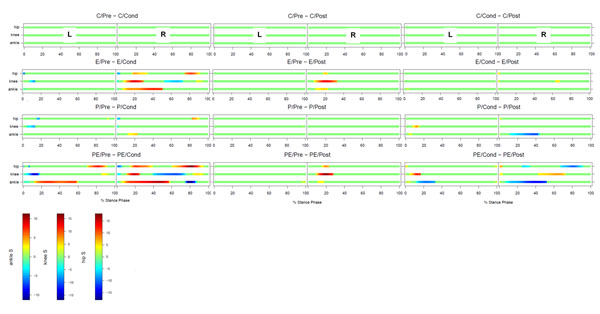
Summary of the FANOVAs. Colours, other than green, indicate between-condition differences. For example, under the effusion condition, hip moment was less for the condition measurement than for the precondition measurement (i.e., experimental knee effusion resulted in a decreased the ankle dorsi-flexion moment at initial contract. L: left (uninvolved), R: right (involved) C: control, E: effusion, P: pain, PE: pain+effusion Pre: precondition, Cond: condition, Post: postcondition

The FANOVAs detected between-session differences for the involved (right) and uninvolved legs (left; Figure 1). The three most important observations are (1) both decreased and increased joint moments were observed during stance phase in all joints, (2) the uninvolved leg was also affected, (3) isolated joint effusion appears to play wider role in joint moment alterations compared to isolated pain, and (4) a combination of pain and joint effusion resulted in a summative effect.

## Conclusion

Stimulation of the receptors specific to joint pressure appears to cause higher impact on alterations in sagittal plane joint moment compared to the nociceptor stimulation. Simultaneous knee joint pain and effusion produced a summative effect on sagittal plane joint moments. Since knee joint effusion and pain are common symptoms in knee joint injuries, both variables should be controlled in acute and chronic phase of rehabilitation in order to avoid altered joint moments.
